# Legislation coverage for child injury prevention in China

**DOI:** 10.2471/BLT.14.139998

**Published:** 2015-01-30

**Authors:** Li Li, Robert Scherpbier, Jing Wu, Xu Zhu, Wei Zhang, Lin Zhang, Xin Gao, Jiesi Luo, Guoqing Hu

**Affiliations:** a Department of Epidemiology and Health Statistics, School of Public Health, Central South University, 110 Xiangya Road, Changsha, 410078, China.; b Section of Health and Nutrition and Water, Environment and Sanitation, United Nations Children’s Fund, Beijing, China.; c Chinese Center for Disease Control and Prevention (China CDC), Beijing, China.

## Abstract

**Objective:**

To examine the extent to which effective interventions to prevent unintentional child injury are reflected in the laws and regulations of China.

**Methods:**

We focused on the six common causes of fatal child injuries – drowning, road traffic injury, falls, poisoning, burns and suffocation. We investigated 27 interventions recommended by the United Nations Children’s Fund, the World Health Organization or the European Child Safety Alliance. We searched China National Knowledge Infrastructure and Lawyee for Chinese legislations using keywords and synonyms for the 27 interventions. We reviewed the identified legislations for statements specifying the responsible implementation department.

**Findings:**

Seven national laws, nine regulations of the State Council and 46 departmental regulations were found to relate to at least one of the interventions. Although seven of the 27 internationally recommended interventions were covered by Chinese laws, 10 were not covered by any current Chinese law or regulation. None of the interventions against drowning and falls that we investigated was covered by national laws. The implementation responsibilities for effective interventions were either not specified or were assigned to multiple governmental departments in 11 or 20 legislative documents, respectively.

**Conclusion:**

In Chinese laws and regulations, interventions proven to prevent major causes of unintentional child injuries are underrepresented and the associated implementation responsibilities are often poorly defined. China should include all such interventions in laws and regulations, and assign implementation responsibility for each to a single department of the national government.

## Résumé

**Objectif:**

Examiner dans quelle mesure les interventions efficaces de prévention des traumatismes involontaires chez l'enfant sont reflétées dans les lois et les réglementations en Chine.

**Méthodes:**

Nous nous sommes concentrés sur les six causes communes de traumatismes mortels chez l'enfant – la noyade, les accidents de la route, les chutes, l'empoisonnement, les brûlures et la suffocation. Nous avons étudié 27 interventions recommandées par le Fonds des Nations Unies pour l'enfance, l'Organisation mondiale de la Santé ou l'Alliance européenne pour la sécurité de l'enfant. Nous avons effectué une recherche sur les lois chinoises dans l'Infrastructure des connaissances nationales de la Chine et Lawyee en utilisant les mots-clés et les synonymes pour les 27 interventions. Nous avons passé en revue les législations identifiées pour chercher les déclarations spécifiant le service de mise en œuvre responsable.

**Résultats:**

Nous avons trouvé 7 lois nationales, 9 réglementations du Conseil d'état et 46 réglementations départementales en relation avec au moins 1 des interventions. Bien que 7 des 27 interventions recommandées internationalement étaient couvertes par les lois chinoises, 10 n'étaient couvertes par aucune loi ou réglementation actuellement en vigueur en Chine. Aucune des interventions contre la noyade et les chutes que nous avons étudiées, n'était couverte par les lois nationales. Les responsabilités de la mise en œuvre des interventions efficaces étaient soit non spécifiées soit affectées à plusieurs services gouvernementaux dans 11 ou 20 documents législatifs, respectivement.

**Conclusion:**

Dans les lois et réglementations chinoises, les interventions qui ont fait leur preuve dans la prévention des causes majeures des traumatismes involontaires chez l'enfant, sont sous-représentées et les responsabilités de mise en œuvre associées sont souvent mal définies. La Chine devrait inclure toutes ces interventions dans ses lois et réglementations, et affecter la responsabilité de la mise en œuvre de chaque intervention à un seul service du gouvernement national.

## Resumen

**Objetivo:**

Examinar en qué grado contemplan las leyes y normativas de China las intervenciones efectivas para prevenir lesiones accidentales de niños.

**Métodos:**

El estudio se centró en las seis causas comunes de lesiones mortales infantiles: ahogamiento, lesiones por accidentes de tráfico, caídas, envenenamiento, quemaduras y asfixia. Se investigaron 27 intervenciones recomendadas por el Fondo para la Infancia de las Naciones Unidas, la Organización Mundial de la Salud o la Alianza Europea para la Seguridad Infantil. A continuación, se realizaron búsquedas de legislaciones de China en las bases de datos China National Knowledge Infrastructure y Lawyee usando palabras claves y sinónimos de las 27 intervenciones. Por último, se revisaron las legislaciones identificadas a fin de buscar declaraciones que especificaran el departamento responsable de la aplicación.

**Resultados:**

Se halló que siete leyes nacionales, nueve normativas del Consejo de Estado y 46 normativas departamentales estaban relacionadas con al menos una de las intervenciones. Aunque las leyes de China abarcaban siete de las 27 intervenciones que se recomiendan a nivel internacional, diez de ellas no estaban incluidas en ninguna normativa o ley actual de China. Las leyes nacionales no abarcaban ninguna de las intervenciones contra ahogamientos y caídas investigadas. Las responsabilidades de aplicación de intervenciones efectivas no estaban especificadas o estaban asignadas a varios departamentos gubernamentales en 11 o 20 documentos legislativos, respectivamente.

**Conclusión:**

En las leyes y normativas de China, las intervenciones que han demostrado la prevención de lesiones infantiles no intencionadas están subrepresentadas y las responsabilidades de aplicación asociadas a menudo tienen una definición vaga. China debe incluir todas estas intervenciones en las leyes y normativas, así como asignar la responsabilidad de aplicación de cada una a un único departamento del gobierno nacional.

## ملخص

**الغرض:**

دراسة مدى انعكاس التدخلات الفعالة لوقاية الأطفال من الإصابات غير المتعمدة على القوانين واللوائح في الصين.

**الطريقة:**

قمنا بالتركيز على الأسباب الشائعة الستة لإصابات الأطفال المميتة - الغرق والإصابات الناجمة عن حوادث المرور وحوادث السقوط والتسمم والحروق والاختناق. وقمنا بتحري 27 تدخلاً أوصى بها صندوق الأمم المتحدة للطفولة (اليونيسيف)، أو منظمة الصحة العالمية، أو التحالف الأوروبي لسلامة الأطفال. وأجرينا بحثاً في البنية الأساسية المعرفية الوطنية في الصين وقاعدة بيانات Lawyee للتشريعات الصينية باستخدام كلمات أساسية ومرادفات للتدخلات السبعة والعشرين. وقمنا باستعراض التشريعات المحددة للعبارات التي تحدد إدارة التنفيذ المسؤولة.

**النتائج:**

تبين وجود صلة بين سبعة قوانين وطنية وتسع لوائح خاصة بمجلس الدولة و46 لائحة إدارية وبين أحد التدخلات على الأقل. وعلى الرغم من تغطية القوانين الصينية سبعة من التدخلات السبع والعشرين الموصى بها على الصعيد الدولي، لم نجد تغطية من أي قانون أو لائحة في الصين في الوقت الراهن لعدد 10 تدخلات. ولم تكن هناك تغطية من القوانين الوطنية لأي من التدخلات لمكافحة الغرق وحوادث السقوط التي قمنا بتحريها. وكانت مسؤوليات التنفيذ للتدخلات الفعالة إما غير محددة أو تم تخصيصها لإدارات حكومية متعددة في 11 أو 20 وثيقة تشريعية، على التوالي.

**الاستنتاج:**

تعاني التدخلات التي ثبت توقيها للأسباب الرئيسية لإصابات الأطفال غير المتعمدة في القوانين واللوائح في الصين من سوء التمثيل، وغالباً ما تعاني مسؤوليات التنفيذ ذات الصلة بها من سوء التحديد. وينبغي أن تقوم الصين بإدراج جميع هذه التدخلات في القوانين واللوائح وتخصيص مسؤولية التنفيذ عن كل تدخل إلى إدارة واحدة في الحكومة الوطنية.

## 摘要

**目的:**

调查中国法律法规在多大程度上反映预防儿童非故意伤害的有效干预措施。

**方法:**

我们把重点放在六种常见的致命儿童受伤原因：溺水、道路交通伤害、跌落、中毒、烧伤和窒息。我们研究了由联合国儿童基金会、世界卫生组织和欧洲儿童安全联盟建议的27种干预措施。我们使用这27种干预措施的关键字和同义词在中国国家知识基础设施（中国知网）和北大法意进行检索。我们分析了被识别法律法规有关干预执行的部门职责分工阐述。

**结果:**

发现7项国家法律、9项国务院规定和46项部门条例与至少一种干预措施相关。虽然国际推荐的27种干预措施中在中国法律中包含了7种，但有10种措施在当前中国任何法律或法规中都没有涉及。国家法律没有涉及我们所调查的任何防溺水和防跌落干预措施。有效干预措施的实施责任在11个立法文件未明确指定，在20个立法文件中被分派给多个政府部门。

**结论:**

在中国的法律法规中，已被证明能预防儿童非故意伤害主因的干预并未得到充分体现，相关实施责任常常界定不清。中国应该在法律法规中包括所有这类干预措施，并将每项实施责任指派到单一的国家政府部门。

## Резюме

**Цель:**

Изучить, насколько эффективные мероприятия по предотвращению неумышленного детского травматизма детей отражены в законодательных и нормативных актах Китая.

**Методы:**

Основное внимание было сосредоточено на шести общих причинах смертельных детских травм: утопление, дорожно-транспортный травматизм, падения, отравления, ожоги и асфиксия. Были исследованы 27 мероприятий, рекомендованных Детским фондом Организации Объединенных Наций, Всемирной организацией здравоохранения или Европейским альянсом за безопасность детей. По базе данных «Китайская национальная инфраструктура знаний и права» проводился поиск китайских законодательных актов по ключевым словам и синонимам 27 мероприятий. Был проведен обзор выбранных законов, содержащих формулировки, указывающие на подразделение, ответственное за реализацию данных мероприятий.

**Результаты:**

Было установлено, что 7 национальных законов, 9 нормативных документов Государственного совета и 46 ведомственных инструкций относились по крайней мере к одному из исследуемых мероприятий. Несмотря на то, что 7 из 27 мероприятий, рекомендованных на международном уровне, предусматривались китайским законодательством, 10 из них не предусматривались никаким действующим китайским законом или иным нормативным актом. Ни одно из исследованных мероприятий по борьбе с утоплением и падением не предусматривалось национальным законодательством. Сведения об ответственном за реализацию эффективных мероприятий либо отсутствовали, либо ответственность распределялась между многочисленными подразделениями правительства соответственно в 11 из 20 законодательных актов.

**Вывод:**

В законодательных и нормативных актах Китая мероприятия, которые гарантированно предотвращают основные причины неумышленного детского травматизма, представлены мало, а совместная ответственность за исполнение таких мероприятий зачастую недостаточно определена. Китаю следует включить все подобные мероприятия в законодательные и нормативные акты и назначить ответственным за их исполнение конкретное подразделение национального правительства.

## Introduction

Child injuries are a public health problem in China. In 2010 – according to Global Burden of Disease estimates – approximately 86 000 individuals aged 0–19 years suffered fatal injuries in China.^1^ In 2008, the *World report on child injury prevention* listed several interventions that had been found effective against unintentional child injuries – e.g. child restraints in vehicles, cycling helmets, pool fencing and flotation devices – and encouraged low- and middle-income countries to adopt such interventions.^2^ However, many of these interventions have yet to be widely implemented in China^3^^,^^4^ – mainly because they are not mandated in national laws or regulations or because responsibility for their implementation has not been clearly assigned to one or more specific governmental departments.

Legal requirements and prohibitions can drive behavioural and environmental changes that can reduce the risk of injury.^5^ There is substantial evidence – albeit mainly from high-income countries – to prove that legislative strategies can be effective in reducing child injuries caused by road traffic, drowning, burns, falls, poisoning or suffocation.^2^^,^^6^ Between 1994 and 2003, for example, the rate of head injuries among people younger than 18 years decreased by 54% in those Canadian provinces that had legislation mandating helmet use for young cyclists but only by 33% in other Canadian provinces.^7^ In New York City, United States of America (USA), legislation requiring landlords to install window guards in all rented properties led to a 96% decrease in the number of children who were seen at hospitals following unintentional falls from windows.^8^

Surprisingly, many interventions known to reduce child injury have not been widely covered by legislation. When investigating legislation covering 10 interventions against child injury in 29 member countries of the Organisation for Economic Co-operation and Development, it was found that none of the 29 countries had legislation covering all 10 interventions. Only seven of the countries – Australia, Canada, Iceland, New Zealand, Norway, Sweden and the USA – had legislation covering at least seven of the interventions.^9^ The World Health Organization (WHO) recently reported that only 28 countries have adequate laws to reduce road traffic injuries by reducing traffic speeds and drink-driving and increasing the use of helmets, seat-belts and child restraints.^10^

The main aims of the present study were to determine which of a set of interventions to prevent child injury were covered by the laws and regulations of China and whether the implementation of such interventions had been assigned to specific governmental departments.

## Methods

### Selected injury-related causes

We focused on the most common causes of fatal unintentional child injuries in China. In 2010, according to Global Burden of Disease estimates,^1^ drowning, road traffic injury, falls, poisoning and burns together accounted for about 73% of all injury-induced deaths among Chinese individuals aged 0–19 years. We therefore investigated these five causes and suffocation. Suffocation was included because, in China in 2010, it was associated with 32% of injury-induced deaths in urban areas and 52% of injury-induced deaths in rural areas of children younger than 1 year.^11^

### Interventions

We investigated the 24 interventions that, according to the *World report on child injury prevention*,^2^ were effective against the five causes that we chose from the Global Burden of Disease. These interventions had all been investigated in robust studies and found to be effective in other countries.^2^ However, as suffocation was not considered, we also investigated three interventions that are known to be effective against child suffocation and are recommended by the European Child Safety Alliance (Table 1).^6^

**Table 1:** Legislative coverage of interventions against the main causes of fatal unintentional child injury, China, 2013

Cause and intervention	Number of times covered by:		
	Law	Regulation issued by State Council	Departmental regulation
**Drowning**			
Removing or covering water hazards	0	1	2
Fencing around swimming pools	0	0	1
Wearing of personal flotation devices	0	0	0
Ensuring immediate resuscitation	0	1	5
**Road traffic injury**			
Introducing and enforcing minimum age for drinking of alcoholic drinks	2	0	1
Setting and enforcing blood alcohol limits for novice drivers, with zero tolerance for offenders	0	0	0
Using appropriate child restraints and seat-belts	0	1	1
Wearing motorcycle and bicycle helmets	1	0	4
Forcing a reduction of speed around schools and residential and play areas	0	1	2
Separating different types of road users	1	4	3
Introducing and enforcing use of daytime running lights for motorcycles	0	0	0
Introducing graduated systems for driver licensing	0	1	1
**Falls**			
Implementing multifaceted community programmes such as “Children Can’t Fly”^8^	0	0	0
Redesigning nursery furniture and other products	0	0	6
Establishing playground standards for the depth of appropriate surface material, height of equipment and maintenance	0	0	5
Legislating for window guards	0	0	3
**Poisoning**			
Removing toxic agents	1	2	7
Child-resistant packaging of medicines and poisons	0	0	0
Packaging drugs in non-lethal quantities	0	0	0
Establishing poison control centres	1	0	2
**Burns**			
Setting and enforcing laws on smoke alarms	1	0	5
Developing a standard for child-resistant lighters	0	0	0
Setting maximum domestic water temperatures	0	0	0
Treating patients at dedicated burn centres	0	0	0
**Suffocation**			
Product modification	1	0	1
Banning of latex balloons, inedible material in food products and pull cords on window coverings	0	0	0
Product warning labels	0	0	3

### Data sources

In China, a law is defined as a legislative document issued by the Standing Committee of the National People’s Congress.^12^ In addition to laws, China also issues regulations of the State Council – i.e. legislative documents, issued by the State Council, that cover the implementation of laws and the matters that Article 89 of the Chinese constitution requests.^12^

China also has legislative documents issued by ministries and commissions under the State Council. These so-called departmental regulations cover the implementation of laws, regulations of the State Council and other orders made by the State Council.^12^

Of the three types of legislative documents issued in China, laws have the strongest legislative power and departmental regulations have the least.^12^

We searched the China National Knowledge Infrastructure^13^ and Lawyee^14^ – i.e. the two most commonly used academic data sets for legislative documents in China – for laws or regulations covering any of the 27 interventions of interest (Table 1).

### Search

We used a three-step approach to develop search words or terms for each intervention of interest. First, we split the name of each intervention into keywords, assuming that various combinations of those keywords could reflect the intervention’s general concept. Second, we expanded the pool of keywords for each intervention to include synonyms and near-synonyms that might be used in laws and regulations. We searched the Handian,^15^ Hanwendadian^16^ and Iciba^17^ online dictionaries for relevant synonyms and near-synonyms. Finally, we searched the two legislative data sets for relevant combinations of the keywords and their synonyms and near-synonyms. If a search for a particular word or term did not yield a result, we excluded that word or term. We were left with a list of 484 search words or terms that could each be linked to a Chinese law or regulation (available from authors).

**Fig. 1:**
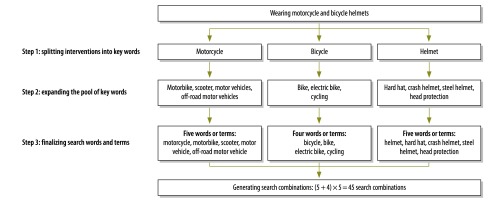
An example of the generation of search words and combinations for Chinese laws and regulations to prevent child injuries

As an example, one of the interventions that we investigated was entitled “the wearing of motorcycle and bicycle helmets”. We separated the intervention title into three independent keywords: “motorcycle”, “bicycle” and “helmets”. We then found five synonyms or near-synonyms for “motorcycle”, four for “bicycle” and five for “helmets”. We combined each of the nine search words or terms for “motorcycle” or “bicycle” with each of the five search words or terms for “helmets” to give 45 search combinations (Fig. 1). We then searched the legislative data sets for each search combination.

Between 20 August 2013 and 16 December 2013, two researchers searched each data set independently before a third researcher checked the search results. Any discrepancies were discussed by all three individuals until a consensus was reached.

For a legislative document to be included in our final analysis, it had to meet four criteria. It had to be: (i) targeted at one or more of the six causes of child injuries; (ii) related or potentially related to at least one of the 27 effective interventions; (iii) applicable to individuals younger than 18 years; and (iv) be in force in October 2013. Each of the two researchers searching the data sets categorized each document as fully relevant, partially relevant or irrelevant. Any document assigned different categories by the two researchers or categorized as only partially relevant by at least one searcher was discussed in group discussions – involving the two searchers and the checker – until it could be categorized as relevant or irrelevant to our final analysis.

### Implementation responsibility

In any country, although the private sector, nongovernmental organizations and advocacy groups may also be involved, governmental departments should take the leading role in the prevention of both unintentional injury and violence.^18^ We therefore studied the implementation responsibility – if any – indicated in the legislative documents included in our final analysis. In group discussions, we categorized the statement of responsibility assignment in a legislative document as “clear” – if it specified one or more departments as responsible and clearly stated the duties of individual departments. If the document mentioned the names of two or more departments but did not specify their duties, or only stated that implementation was the responsibility of relevant departments but did not specify the names and duties of those departments or did not mention governmental departments at all, we categorized the statement of responsibility assignment as “unclear”.

## Results

In October 2013, the China National Knowledge Infrastructure covered all of the 242 laws then in force in China – and held the details of 511 documents on laws, 6699 on regulations of the State Council and 98 604 on departmental regulations. The corresponding values for the Lawyee data set were 240 laws and 1344, 6060 and 125 635 documents, respectively. We assumed that the combination of the China National Knowledge Infrastructure and Lawyee covered all regulations that were in force at the time of our searches.

We identified 62 legislative documents – seven national laws, nine regulations of the State Council and 46 departmental regulations – that we considered to be relevant to at least one of the 27 interventions that we were investigating and included in the final analysis. Some interventions are covered by multiple laws or regulations simultaneously.

**Fig. 2:**
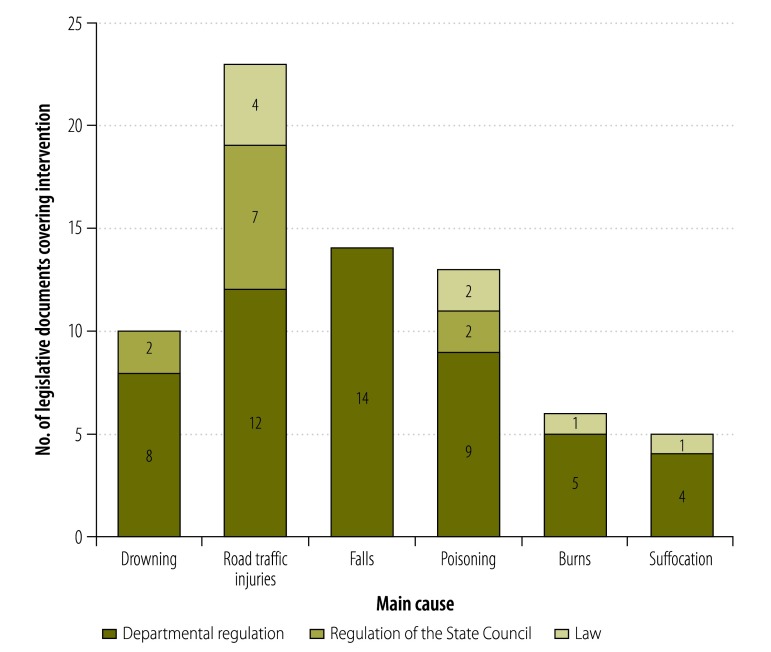
Coverage of interventions against unintentional child injury in laws and regulations, China, 2013

Notes: Coverage is shown separately for each of six main causes of injury. Some interventions were covered concurrently by multiple laws or regulations.

Interventions against child deaths caused by road traffic injury were covered by four laws, seven regulations of the State Council and 12 departmental regulations (Fig. 2). Interventions against falls were also relatively well covered by legislative documents but no law covered interventions against unintentional child deaths by drowning. Of the 27 interventions that we investigated, 10 were not covered by any Chinese laws and regulations at the time of our searches, seven were covered by laws – including one intervention covered by two laws simultaneously – and seven were covered by regulations of the State Council (Table 1).

**Fig. 3:**
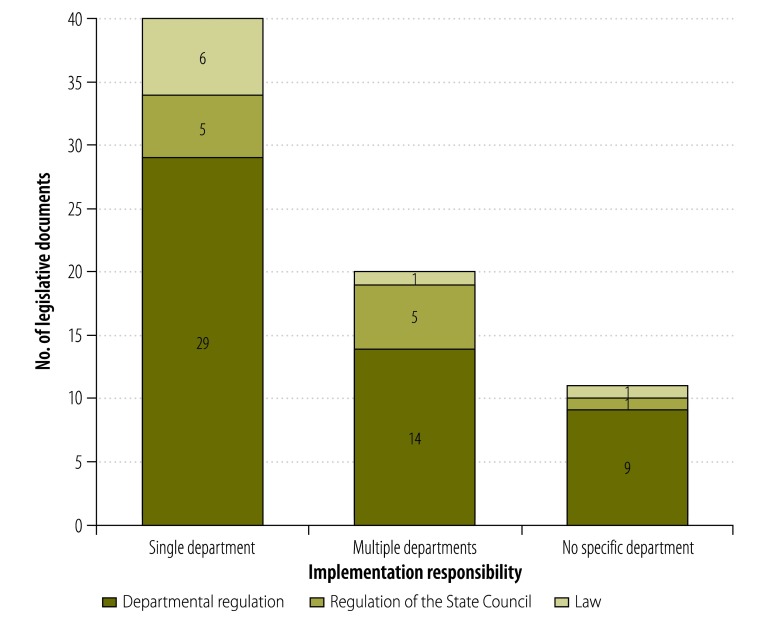
Assignment of implementation responsibility in legislative documents covering interventions against unintentional child injury, China, 2013

Notes: The numbers of legislative documents are shown separately according to the number of specified departments made responsible for the implementation of the legislation. Some interventions were covered concurrently by multiple laws or regulations.

One law, one regulation of the State Council and nine departmental regulations failed to assign the implementation of interventions to any specific departments. Other legislative documents assigned such implementation responsibilities to one or more specified departments (Fig. 3).

## Discussion

In China in 2013, we found that 10 of 27 effective interventions against injury-induced child mortality had not been covered by any laws or regulations. The 17 interventions that were covered by some legislative documentation were more likely to be covered by departmental regulations than by more powerful laws or regulations of the State Council. In addition, many of the associated legislative documents were weakened by their failure to assign responsibilities for the implementation of interventions to named governmental departments.

Our study did have some limitations. For example, it was impossible to determine what percentage of current regulations of the State Council and departmental regulations were covered by the two data sets. We could therefore not determine if we missed regulations that covered any of the investigated interventions. Other potential bias may have resulted from the subjective assessment of researchers that was involved in much of our analysis. However, in an attempt to reduce such bias, we adopted a systematic approach to ensure the standardized implementation of each step in our analysis. We focused on effective interventions that had been identified in technical reports published in 2006^6^ or 2008.^2^ We therefore took no account of either interventions that were only found to be effective in the last few years or interventions that are only effective against the minor causes of fatal child injuries. We also ignored the possible coverage of the interventions that we did investigate in local regulations or recommendations within China. In Shanghai, for example, the use of car seats for children has been made mandatory and promoted through the efforts of multiple parties.^19^

There are probably two main reasons why 10 effective interventions are not currently covered by Chinese laws and regulations. First, legislation to cover some interventions may be difficult because the interventions are seen as too expensive to implement nationwide or because the intervention is seen as unnecessary in China. The establishment of dedicated poison control centres and dedicated burns centres, for example, may be perceived as having limited benefit given the existing health-care system in China. Second, any legislation – including that needed to cover interventions that are relatively easy to implement and perceived to be very useful – takes considerable time to develop. The use of child-resistant packaging of medications – which can markedly reduce unintentional child poisoning^20^ – was recommended by a Chinese researcher in 2011 but this recommendation still had no legislative support in China in 2013.^21^

Laws and regulations cannot be implemented effectively when they fail to specify the government departments responsible for their implementation. It was recently reported that regulatory failures are primarily due to gaps in regulatory design or implementation.^22^ Such gaps include the failure to legislate clearly on regulatory function, the failure to assign regulatory organization, insufficient human resources, ambivalence in the roles of regulatory organizations and ineffective coordination between multiple regulatory organizations.

Legislation is only the first step to promote the use of effective interventions. Once laws designed to prevent unintentional child injuries are approved, they are most effective when enforcement is strong. Available evidence suggests that the enforcement of some safety-related laws is inadequate in China. In 2013, for example, WHO graded only one of four interventions related to road traffic injury that are covered by the same Chinese law – i.e. an intervention against drink-driving – as being strongly enforced.^10^ The enforcement of interventions to reduce speeding or increase the use of seat-belts or motorcycle helmets was considered to be much weaker.^10^

## Conclusion

Several effective interventions against the six major causes of fatal unintentional child injuries are not covered by the current laws and regulations of China. Even for such interventions that are covered by laws or regulations, the implementation responsibilities are often poorly defined. The government of China could substantially reduce the risk of unintentional injury for millions of children by revising current laws and regulations – or establishing new ones – to cover more interventions that have proven to be effective. Whenever possible, such interventions should be covered by national laws rather than by regulations. The governmental departments responsible for the execution of the interventions need to be clearly identified in any associated legislation. A strong and clear law is better than several weak and obscure regulations. Legislators should continue to monitor scientific discoveries so that legislation to support any novel and effective interventions can be developed quickly.
